# Tolvaptan in ADPKD Patients With Very Low Kidney Function

**DOI:** 10.1016/j.ekir.2021.05.037

**Published:** 2021-06-09

**Authors:** Vicente E. Torres, Ron T. Gansevoort, Ronald D. Perrone, Arlene B. Chapman, John Ouyang, Jennifer Lee, Hina Japes, Ali Nourbakhsh, Tao Wang

**Affiliations:** 1 Division of Nephrology and Hypertension, Mayo Clinic, Rochester, Minnesota, USA; 2 Division of Nephrology, Universitair Medisch Centrum Groningen, Groningen, The Netherlands; 3 Division of Nephrology, Tufts Medical Center, Boston, Massachusetts, USA; 4 Division of Nephrology, The University of Chicago, Chicago, Illinois, USA; 5 Otsuka Pharmaceutical Development & Commercialization, Inc., Rockville, Maryland, USA; 6 Otsuka Pharmaceutical Development & Commercialization, Inc., Princeton, New Jersey, USA

**Keywords:** autosomal dominant polycystic kidney disease, chronic kidney disease, clinical trials, glomerular filtration rate, *post hoc* analysis, tolvaptan

## Abstract

**Introduction:**

Tolvaptan slowed estimated glomerular filtration rate (eGFR) decline in subjects with autosomal dominant polycystic kidney disease (ADPKD) in TEMPO 3:4 and REPRISE trials. Tolvaptan effects in subjects with eGFR 15 to 24 ml/min per 1.73 m^2^ were not investigated. This *post hoc* analysis retrospectively investigated eGFR decline in REPRISE versus an open-label, phase 3b extension trial (open-label extension [OLE] NCT02251275) in subjects who received placebo in REPRISE and tolvaptan in OLE with eGFR 15 to 24 and 25 to 29 ml/min per 1.73 m^2^, respectively.

**Methods:**

One data subset comprised subjects with OLE baseline eGFR 15 to 29 ml/min per 1.73 m^2^ who had received placebo in REPRISE and began tolvaptan in OLE. The second comprised subjects who had received tolvaptan in REPRISE and were matched to REPRISE placebo-treated subjects for REPRISE baseline characteristics. Annualized eGFR slopes in REPRISE versus OLE were compared within the REPRISE placebo (i.e., placebo vs. tolvaptan treatment) and tolvaptan (i.e., 2 periods of tolvaptan treatment) subsets.

**Results:**

Mean annualized eGFR slopes (ml/min per 1.73 m^2^) during tolvaptan treatment in OLE versus placebo treatment in REPRISE were −3.4 versus −5.2 for subjects with OLE baseline eGFR 15 to 29 (difference, 1.7; *P* < 0.001), −3.6 versus −5.4 with baseline eGFR 15 to 24 (difference, 1.8; *P* < 0.001), and −3.3 versus −4.9 with baseline eGFR 25 to 29 (difference, 1.6; *P* < 0.001). In REPRISE tolvaptan subjects who continued tolvaptan in OLE, treatment effect was maintained (no difference between mean annualized eGFR slopes).

**Conclusion:**

Initiating or maintaining tolvaptan therapy significantly delayed eGFR decline in subjects with baseline eGFR 15 to 24 and 25 to 29 ml/min per 1.73 m^2^.

## Graphical abstract

**Figure:**
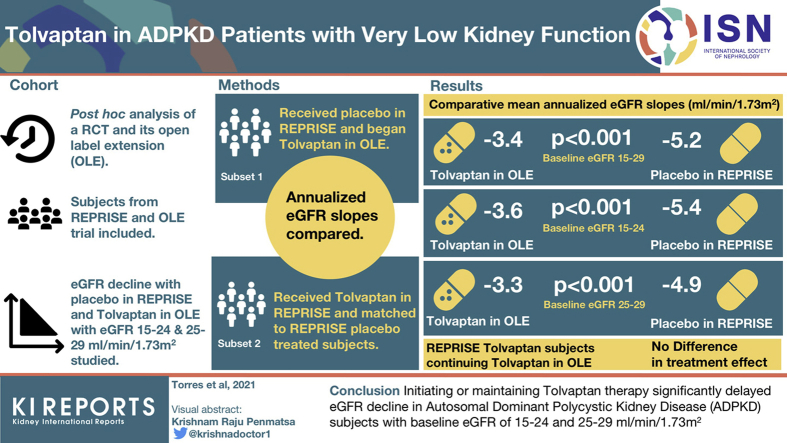

Autosomal dominant polycystic kidney disease (ADPKD) is a common inherited disorder characterized by a progressive increase in the number and size of kidney cysts leading to reduced glomerular filtration rate (GFR) and eventual kidney failure in most affected individuals.^1^^,^^2^ Tolvaptan is a selective arginine vasopressin type 2 receptor antagonist found to slow the progression of kidney function decline in ADPKD.^3^^,^^4^

Two multicenter, randomized, placebo-controlled trials evaluated the safety and efficacy of tolvaptan in treating subjects at different stages of ADPKD. The TEMPO 3:4 trial (NCT00428948, ClinicalTrials.gov) enrolled subjects with early to midstage chronic kidney disease (CKD) (baseline eGFR clearance ≥ 60 ml/min per 1.73 m^2^).^3^^,^^5^ Meeting the primary end point of kidney size, tolvaptan was associated with a 2.8% per year increase in kidney volume compared with a 5.5% per year increase in the placebo group (*P* < 0.001). Tolvaptan was also associated with a slower decline in kidney function, as measured by reciprocal of serum creatinine level, compared with placebo, –2.61 vs. −3.81 (mg/ml)^–^^1^ per year (*P* < 0.001). The treatment effect was +1.20 (mg/ml)^–^^1^ per year (95% confidence interval: 0.62–1.78; *P* < 0.001). A second trial, REPRISE (NCT02160145), confirmed and expanded these observations by evaluating the efficacy and safety of tolvaptan in a 12-month period in subjects at more advanced stages of disease (baseline eGFR 25–65 ml/min per 1.73 m^2^).^4^^,^^6^ In a subgroup analysis stratifying REPRISE subjects according to eGFR, tolvaptan was found to significantly delay eGFR decline in subjects at eGFR 45 to 59, 30 to 44, and 25 to 29; treatment effects were 2.36 ml/min per 1.73 m^2^ (*P* < 0.001), 0.78 ml/min per 1.73 m^2^ (*P* < 0.008), and 0.81 ml/min per 1.73 m^2^ (*P* = 0.02), respectively.^4^ Given this trial excluded subjects with baseline eGFR < 25 ml/min per 1.73 m^2^, conclusions could not be drawn on tolvaptan efficacy in late CKD G4 (eGFR 15–29). Subjects who completed TEMPO 4:4, REPRISE, or other tolvaptan trials could enroll in this prospective, multinational, OLE safety trial (NCT02251275) to evaluate the long-term safety of tolvaptan in subjects with ADPKD.^7^ The objective of this *post hoc* analysis was to evaluate the safety profile and efficacy of tolvaptan in the enrolled participants from the REPRISE trial who at the time of enrollment in the OLE trial had a baseline eGFR 15 to 29 ml/min per 1.73 m^2^, with specific interest in evaluating eGFR decline in subjects with baseline eGFR 25 to 29 and 15 to 24 ml/min per 1.73 m^2^.

## Methods

### Participants and Study Design

This retrospective analysis included subjects who had participated in REPRISE and OLE trials. In REPRISE, subjects were administered tolvaptan at daily morning and afternoon doses of 60 and 30 mg or 90 and 30 mg, respectively.^6^ Subjects from REPRISE enrolled in the OLE were initiated on tolvaptan at a split dose of 45/15 mg with upward titration every 3 to 4 days to 60/30 mg or 90/30 mg per day according to tolerability.^7^ In REPRISE, creatinine was measured monthly,^6^ and in OLE, it was measured at monthly visits for subjects < 18 months on tolvaptan and every 3 months for subjects > 18 months on tolvaptan.^7^

Criteria for inclusion in the initial subject selection for this *post hoc* analysis were subjects who had a diagnosis of ADPKD, were 18 years of age or older, had an OLE baseline eGFR of 15 to 29 ml/min per 1.73 m^2^ (CKD G4), had been randomized to the placebo group in the REPRISE trial, and had received at least one dose of tolvaptan in the tolvaptan OLE. Although the OLE trial included subjects from other tolvaptan trials, only those who had previously been enrolled in the REPRISE trial were selected for inclusion in this analysis. Subjects from the OLE trial, who fulfilled the *post hoc* analysis inclusion criteria described previously, were identified as the REPRISE placebo subset. These subjects were then matched 1:1 with those who had been randomized to the REPRISE trial tolvaptan arm (REPRISE tolvaptan subset). In contrast to subjects in the REPRISE placebo subset, those in the REPRISE tolvaptan subset may not have had an eGFR of 15 to 29 at the OLE trial baseline. Subjects in the REPRISE tolvaptan subset were matched to those in the REPRISE placebo subset at the REPRISE baseline, which included subjects with eGFR 25 to 65 ml/min per 1.73 m^2^. Tolvaptan treatment during REPRISE may have reduced their eGFR decline, such that eGFR was >30 ml/min per 1.73 m^2^ at the OLE baseline.

Matching was performed using both the CKD stage and eGFR to ensure that the CKD stage matched exactly, and then matching was performed based on propensity scores for REPRISE baseline characteristics of gender, eGFR, and age (REPRISE tolvaptan subset). As the matched data set was intended to provide a control comparison of subjects treated with tolvaptan during the REPRISE trial, subject propensity score matching was performed to avoid bias in selecting subjects for comparison. The covariates of age and eGFR were treated as variables in the propensity score model that created the propensity score for each subject. The propensity score is the probability of assigning a subject to the tolvaptan group conditional on a set of observed baseline covariates of age, eGFR, sex, and CKD stage. It was computed by fitting a logistic regression model with a response variable of assignment to the tolvaptan group and the set of baseline covariates. The match was performed requesting an exact match on CKD stage and using the propensity score for match within a CKD stage without regarding the actual values of age or eGFR. SAS PROC PSMATCH was used to perform the propensity score match with the default option of optimal matching of one tolvaptan subject to each subject in the placebo group. The rationale for including CKD stage as a covariate in the calculation of the propensity score is that CKD stage was related to the treatment assignment in REPRISE, as randomization stratification was based on eGFR baseline of at least 45 ml/min per 1.73 m^2^ or not. Inclusion of CKD stage as a variable in the model may still have impact on determination of regression coefficients in the logistic model, even though the contribution of a CKD stage would be cancelled in the propensity score for all subjects with the same CKD stage owing to exact match. In this way, each subject from the placebo group was matched by propensity score to the nearest record in the tolvaptan group that was in the same CKD stage of the placebo subject and had not yet been matched. Thus, the *post hoc* analysis data set comprised matched pairs, each pair with a REPRISE placebo and a REPRISE tolvaptan subject. For the REPRISE placebo subjects only, of each matched pair, the key focus was to compare their annualized eGFR slopes (first 12 months only) in the OLE trial with their annualized eGFR slopes in the 12-month REPRISE trial. eGFR slopes were thus compared in the same subjects during placebo (REPRISE) and tolvaptan exposure (OLE). For the REPRISE tolvaptan subjects only, of each matched pair, the control analysis was to compare their annualized eGFR slopes (first 12 months only) in the OLE trial with their annualized eGFR slopes in the 12-month REPRISE trial. Annualized eGFR slopes were thus compared in the same subjects during tolvaptan exposure in both trials.

The control analysis was performed to reveal whether observed differences in eGFR decline in REPRISE compared with OLE for the REPRISE placebo subset were attributable to a tolvaptan treatment effect or alternatively to a plateau in eGFR decline in the OLE trial associated with late-stage kidney disease, as suggested by data from the Modification of Diet in Renal Disease.^8^

The overall data set comprising matched subjects who had received placebo or tolvaptan in REPRISE was further divided into 2 mutually exclusive eGFR subgroups (eGFR 15–24 and eGFR 25–29) for additional analysis. Effects of tolvaptan in subjects with eGFR of 25 to 29 have previously been evaluated in REPRISE.^6^ The tolvaptan OLE trial however included subjects with an eGFR of <25 ml/min per 1.73 m^2^; therefore, this *post hoc* analysis was able to include subjects with lower kidney function than previously evaluated in randomized controlled trials. Informed consent procedures and adherence to ethical standards in the REPRISE and OLE trials have been described.^4^^,^^7^

### Variables Evaluated

The variables evaluated in this *post hoc* analysis included baseline characteristics and demographic information of 2 subject data sets, treatment duration, adverse event profile, and eGFR change from baseline over time. eGFR was calculated using the CKD Epidemiology Collaboration equation.^9^

### Statistical Analysis

Comparisons in this analysis were made by linear mixed model, which included fixed-effect terms of subject, treatment, time, subject–time interaction, and treatment–time interaction, with intercept and time as random effects. Similarly, annualized eGFR slopes in the REPRISE tolvaptan subset between the periods of tolvaptan treatment in OLE and REPRISE were compared. Two sensitivity analyses were performed based on propensity score.

Analysis of mixed-model repeated measurements was applied to subjects who had a baseline eGFR 15 to 29 ml/min per 1.73 m^2^ in the OLE trial, received at least 1 dose of tolvaptan treatment in the OLE trial, and were randomized to the placebo group in the REPRISE trial (REPRISE placebo subset), including to their matched subjects randomized to the tolvaptan group in the REPRISE trial (REPRISE tolvaptan subset). The mixed-model repeated measurement analysis was conducted using the combined eGFR observations of these subjects from the REPRISE and OLE trials, and the model included fixed-effect terms of treatment, visit, and treatment–visit interaction and covariates of REPRISE eGFR baseline and baseline–visit interaction. Heterogeneous Toeplitz variance–covariance matrix was used to model the within-subject correlation of eGFR observations. All adverse events reported during the course of the OLE trial were collected and summarized.

## Results

### Disposition and Baseline Characteristics of Analysis Population

**Figure 1:**
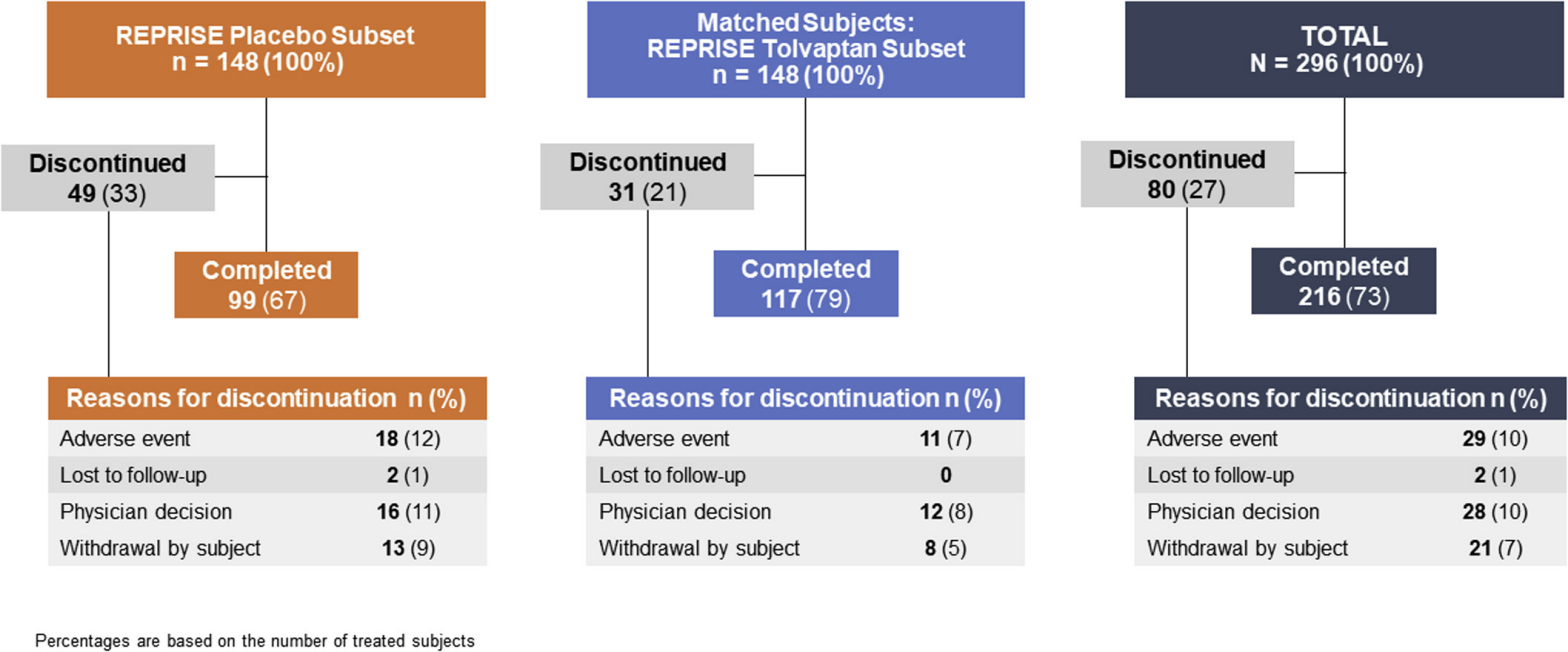
Subject disposition during the first 12 months of tolvaptan therapy in OLE trial. OLE, open-label extension.

A total of 157 subjects from the OLE who had received placebo in REPRISE met the inclusion criteria for initial subject selection for this analysis. Among these 157 subjects randomized to placebo in REPRISE, a subset of 148 subjects (REPRISE placebo subset) were identified who could be matched, based on their characteristics at REPRISE baseline, with 148 subjects in the REPRISE tolvaptan arm, who were then defined as the REPRISE tolvaptan subset. Thus, this *post hoc* analysis population comprised 296 matched subjects, or 148 matched pairs, each pair with a REPRISE placebo and a REPRISE tolvaptan subject. Table 1 lists the REPRISE baseline values of the propensity score covariates and the OLE baseline characteristics of both subsets. Most OLE baseline characteristics of subjects in the 2 subsets were comparable to each other and across OLE baseline eGFR subgroups. In both subsets, the percentage of male subjects was slightly higher in the eGFR 15 to 24 subgroup than the eGFR 25 to 29 subgroup. The disposition of each subset during their first 12 months of tolvaptan therapy reveals that the percentage of subjects who discontinued during the first 12 months of treatment in the OLE was higher in the REPRISE placebo subset (33%) than the REPRISE tolvaptan subset (21%) likely owing to a higher percentage of subjects in the REPRISE placebo subset who experienced side effects of tolvaptan in the OLE (Figure 1 and Supplementary Table S1).

**Table 1:** OLE baseline demographics of the study population according to OLE baseline eGFR subgroup

Characteristics	REPRISE placebo subset		REPRISE tolvaptan subset	
	*N* = 148		*N* = 148	
eGFR subgroup^a^	15–24	25–29	15–24	25–29
n	75	73	75	73
REPRISE baseline propensity score covariates				
CKD stage:				
CKD2	0	0	0	0
CKD3	11	51	11	51
CKD4	64	22	64	22
Mean/median eGFR^a^	27.6/27.6	32.9/31.8	27.7/27.4	32.6/32.2
Mean age, y (SD)	47.5 (7.4)	48.7 (8.5)	47.5 (7.6)	50.7 (7.9)
Men, *n* (%)	44 (59)	36 (49)	43 (57)	38 (52)
OLE baseline				
Mean/median eGFR^a^	21.8/22.4	27.5/27.3	24.1/23.9	29.9/28.6
Mean age, y (SD)	49 (7)	50 (9)	49 (8)	52 (8)
Mean height, cm (SD)	173 (12)	172 (9)	176 (10)	173 (12)
Mean weight, kg (SD)	85 (16)	84 (16)	90 (21)	82 (17)
Race, n (%)				
White	71 (95)	66 (90)	69 (92)	66 (90)
Black/African American	2 (3)	5 (7)	3 (4)	4 (6)
Other^b^	2 (3)	2 (3)	3 (4)	3 (4)
Ethnicity, *n* (%)				
Hispanic or Latino	3 (4)	3 (4)	5 (7)	7 (10)
Not Hispanic or Latino	72 (96)	70 (96)	70 (93)	66 (90)

CKD, chronic kidney disease; eGFR, estimated glomerular filtration rate; OLE, open-label extension.

a ml/min per 1.73 m^2^.

b Other includes American Indian, Alaskan Native, or Asian.

### Efficacy

Among all subjects in the overall REPRISE placebo subset, the mean annualized eGFR slopes (ml/min per 1.73 m^2^) during tolvaptan treatment in the OLE trial versus during placebo treatment in REPRISE were −3.4 versus −5.2, respectively, corresponding to a significant treatment effect (1.72, *P* < 0.001) of tolvaptan (Table 2). Similar results were obtained across each of the 2 eGFR subgroups (15–24 and 25–29) (*P* < 0.001 for comparison in each eGFR subgroup) (Table 2). These results suggest that initiating tolvaptan therapy can significantly delay eGFR decline compared with no treatment in subjects with eGFR 15 to 24, including those with eGFR 25 to 29.

**Table 2:** Mean annualized eGFR (CKD-EPI) slope in subjects matched by propensity score according to eGFR subgroup

eGFR 15–29^a^ Overall	*N* = 296					
REPRISE placebo subset						
Overall and eGFR subgroups, *n*		Trial and treatment		Treatment effect^b^		*P* value
		REPRISE placebo slope (SE)	OLE tolvaptan slope (SE)	Difference	95% CI	
Overall (eGFR 15–29)	148	−5.172 (0.260)	−3.430 (0.232)	1.742	1.330–2.154	<0.001
eGFR 15–24	75	−5.444 (0.314)	−3.607 (0.281)	1.838	1.338–2.338	<0.001
eGFR 25–29	73	−4.903 (0.414)	−3.280 (0.367)	1.623	0.970–2.277	<0.001

REPRISE tolvaptan subset						
Overall and eGFR subgroups, *n*		REPRISE tolvaptan, slope (SE)	OLE tolvaptan, slope (SE)	Difference	95% CI	*P* value
Overall (eGFR 15–29)	148	−3.477 (0.238)	−3.400 (0.215)	0.077	−0.295 to 0.448	0.69
eGFR 15–24	75	−3.770 (0.325)	−3.374 (0.299)	0.395	−0.120 to 0.911	0.13
eGFR 25–29	73	−3.166 (0.347)	−3.413 (0.310)	−0.247	−0.782 to 0.288	0.37

CKD-EPI, chronic kidney disease epidemiology collaboration creatinine equation; eGFR, estimated glomerular filtration rate; OLE, open-label extension trial.

Serum creatinine was only by rate blank in OLE; hence, method was changed from enzymatic (primary efficacy) to rate blank in REPRISE for comparison compatibility. Tolvaptan run-in visits of REPRISE were included. Owing to hemodynamic effect, the baseline of OLE was not included.

a ml/min/1.73 m^2^ per year.

b Derived from linear mixed model with terms of subject, treatment, time, interaction of treatment and time, interaction of subject and time, and baseline for intrasubject comparison between REPRISE and OLE. Intercept and time are treated as random effects.

To provide support for the conclusion that the greater rate of eGFR decline for subjects in the REPRISE placebo subset in REPRISE compared with OLE was a tolvaptan treatment effect, the same analysis was performed with subjects in the REPRISE tolvaptan subset. No significant difference was observed between annualized eGFR slopes in subjects from the overall REPRISE tolvaptan subset during REPRISE and OLE trials (eGFR slope −3.4 vs. −3.5; treatment effect of 0.08 ml/min per 173 m^2^, 95% confidence interval: −0.45 to 0.30, *P* = 0.69), suggesting severity plateau of eGFR decline was not a contributing factor to eGFR slope differences between REPRISE and OLE trials observed in subjects from the REPRISE placebo subset (Table 2). Moreover, annualized eGFR slopes were comparable across eGFR subgroups in the REPRISE tolvaptan subset (slopes ranging from −3.8 to −3.2) during the 2 trials and were in the same range as the REPRISE placebo subset, across eGFR subgroups, in the OLE trial. Together, these findings suggest that initiating tolvaptan treatment can delay eGFR decline in subjects with low eGFR and that the delayed rate of decline in eGFR with tolvaptan treatment is constant, regardless of previous tolvaptan treatment, in a 2-year period.

**Figure 2:**
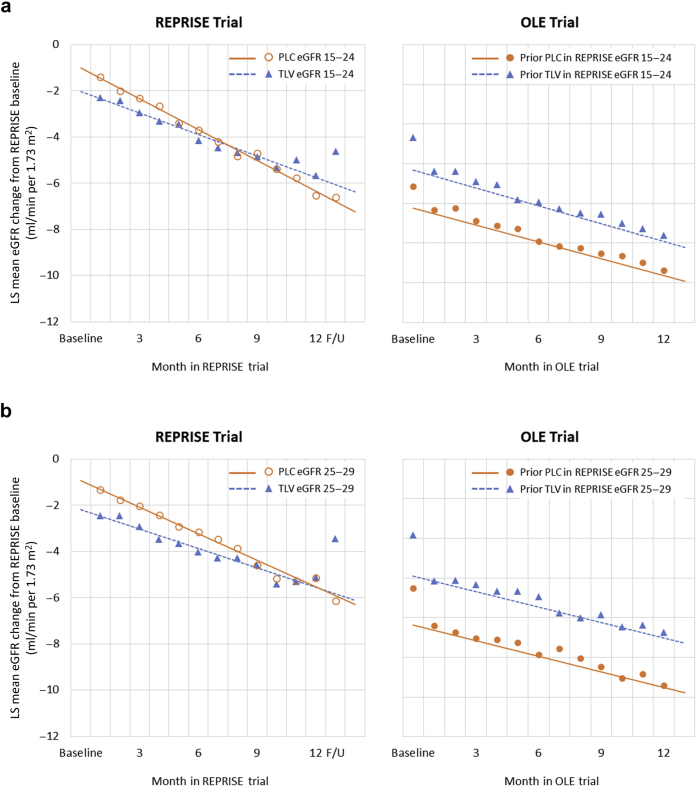
Comparison of eGFR slopes between REPRISE placebo and tolvaptan subsets for the 2 eGFR subgroups (15–24 and 25–29) during the REPRISE and OLE trials. (a, b) Mean change from REPRISE baseline in eGFR (in ml/min per 1.73 m^2^) in 1 year in REPRISE and OLE for matched subjects in OLE baseline eGFR subgroups. All regression lines based on months 1 to 12 data only. Derived from a mixed-model repeated measures analysis with fixed factors of treatment, visit, treatment–visit interaction, baseline value, and baseline–visit interaction as covariates and with a heterogeneous Toeplitz variance–covariance matrix. eGFR, estimated glomerular filtration rate; F/U, follow-up time point; LS, least squares; OLE, open-label extension; PLC, placebo; TLV, tolvaptan.

Mean monthly eGFR changes from the REPRISE baseline in a year were analyzed between REPRISE placebo and tolvaptan subsets for the 2 eGFR subgroups (15–24 and 25–29) in the REPRISE and OLE trials (Figure 2a and b). In the REPRISE trial, for both eGFR subgroups, the eGFR decline slope was steeper in the placebo subset compared with the tolvaptan subset. In contrast, in the OLE trial, for both eGFR subgroups, the eGFR decline slopes for REPRISE placebo and tolvaptan subsets were parallel. Mean eGFR change from baseline for subjects in the REPRISE placebo subset during the first month of the OLE trial was comparable between matched subjects in the REPRISE tolvaptan subset during the first month of the REPRISE trial and that of the OLE trial in both eGFR subgroups, likely owing to the acute hemodynamic effect that occurs after tolvaptan initiation.^4^^,^^10^^,^^11^ After the first month in the OLE trial, eGFR changes from the REPRISE baseline were similar between REPRISE placebo and tolvaptan data sets, for both eGFR subgroups (15–24 and 25–29). A significant difference between previous treatment subsets, favoring the REPRISE tolvaptan subset, was observed between eGFR change from REPRISE baseline at follow-up visit of the REPRISE trial for each eGFR subgroup (1.98 for eGFR subgroup 15–24, *P* < 0.001, and 2.68 for eGFR subgroup 25–29, *P* < 0.001). The tolvaptan treatment effects were maintained for each eGFR subgroup through the 1-year period of the OLE trial (1.78 for eGFR 15–24, *P* = 0.001, and 2.66 for eGFR 25–29, *P* <0.001).

Sensitivity analyses (covariate and treatment weight) based on propensity score confirmed the significant improvement in eGFR decline with tolvaptan treatment in all eGFR subgroups evaluated in this study. Together, these findings reveal that tolvaptan has comparable efficacy in reducing eGFR decline in subjects with later stage kidney disease as in subjects with higher eGFR, regardless of REPRISE exposure to tolvaptan or placebo.

### Safety and Tolerability

Safety data were from the entire 3-year treatment period of the OLE trial. Adverse events in the overall REPRISE and OLE trial populations have been reported elsewhere.^4^^,^^7^ A summary of adverse events during the OLE indicates an even distribution of events (range: 504–647) between the 2 matched subsets (and across the 2 eGFR subgroups; Table 3). The 5 most frequent adverse events in both subsets in the overall eGFR subgroup (15–29) were related to the aquaretic effects of tolvaptan (thirst, polyuria, nocturia) or ADPKD (kidney pain, creatinine increase) (Supplementary Table S1), with a similar pattern for serious treatment-emergent adverse events (Supplementary Table S2). Adverse events related to abnormal liver enzyme tests were infrequent. Hepatic safety was carefully monitored with monthly liver enzyme tests, and adverse events related to abnormal liver enzyme tests were <2.0% (Table 3). Results are consistent with patterns found in the overall trial populations.

**Table 3:** Summary of adverse events according to OLE baseline eGFR subgroup

Subjets and events	REPRISE placebo subset		REPRISE tolvaptan subset	
eGFR^a^	15–24	25–29	15–24	25–29
Subjects treated, *n* (%)^b^	75 (100)	73 (100)	75 (100)	73 (100)
Subject days of drug exposure, *n*	39,992	41,439	43,025	44,948
Subjects with AEs, *n* (%)	68 (91)	70 (96)	69 (92)	71 (97)
AEs, *n*	565	647	504	576
TEAEs, *n*^c^	468	528	438	471
Subjects with serious TEAEs, *n* (%)	12 (16)	16 (22)	22 (29)	16 (22)
Subjects with nonserious TEAEs, *n* (%)	67 (89)	70 (96)	68 (91)	71 (97)
Subjects with severe TEAEs, *n* (%)	9 (12)	11 (15)	15 (20)	10 (14)
Deaths, *n* (%)	1 (1)	0	0	1 (1)

AE, adverse event; eGFR, estimated glomerular filtration rate; OLE, open-label extension; TEAE, treatment-emergent adverse event.

a ml/min per 1.73 m^2^.

b Percentages based on number of subjects treated.

c TEAEs defined as AEs that started after trial drug treatment; or if AE was continuous from baseline and was serious or trial drug related, or resulted in death, discontinuation, interruption, or reduction of trial therapy. Multiple occurrences of TEAEs counted once per MedDRA preferred term.

## Discussion

On the basis of the TEMPO 3:4 results, tolvaptan was approved for rapidly progressive ADPKD in Japan, Canada, the European Union, Switzerland, Nordic countries, South Korea, and Australia. After the publication of the results of REPRISE,^4^ tolvaptan was approved for rapidly progressive disease in the United States and other countries. It was then also recommended in the European Union that the eGFR threshold to initiate tolvaptan treatment be lowered from >45 to 30 ml/min per 1.73 m^2^.^12^

A *post hoc* analysis of TEMPO 3:4 revealed that the treatment effect of tolvaptan slowing the rate of eGFR decline (ml/min per 1.73 m^2^ per year) was not demonstrable in CKD G1 subjects (0.4, *P* = 0.23), was statistically significant in CKD G2 (1.13, *P* < 0.001), and most marked in CKD G3a subjects (1.66, *P* < 0.001). In REPRISE, the treatment effect of tolvaptan was greatest in CKD 3a (2.36, *P* < 0.001) and less in CKD G3b (0.78, *P* = 0.08) and CKD G4 with eGFR ≥ 25 ml/min per 1.73 m^2^ (0.81, *P* = 0.02), raising the concern that administration of tolvaptan could become futile or even detrimental in subjects with an eGFR 15 to 24. The treatment effect in subjects with CKD G4 (eGFR 25–29) in the REPRISE trial was 0.81 ml/min per 1.73 m^2^ compared with 1.62 in this *post hoc* analysis.^6^ Higher treatment effects in this *post hoc* analysis can be attributed to elimination of intersubject variability by the pair-wise comparison. Treatment effects between eGFR subgroups 15 to 24 and 25 to 29 in this analysis were similar. Thus, whether in a randomized, placebo-controlled trial or retrospective subject self-control *post hoc* analysis, tolvaptan treatment effects were observed in subjects with CKD G4.

Until now, there has been no information regarding effects of starting tolvaptan when the eGFR is 15 to 24 ml/min per 1.73 m^2^, including whether it could potentially induce hemodynamic effects or whether the administration of tolvaptan should be discontinued when the eGFR drops below 25 ml/min per 1.73 m^2^. The urgency of this evidence gap is emphasized by the common question in clinical practice as to whether tolvaptan can be initiated in patients with eGFR < 30 and whether and when to discontinue tolvaptan before reaching kidney failure.

This *post hoc* analysis of a large number of subjects with advanced late CKD G4 (ADPKD) revealed that the rate of eGFR decline is significantly reduced when the treatment is changed from placebo in REPRISE to tolvaptan in the OLE trial, not only in the subjects with a baseline eGFR between 25 and 30 ml/min per 1.73 m^2^ but also in those with a baseline eGFR 15 to 24 ml/min per 1.73 m^2^. The observation that annualized eGFR slopes were similar in magnitude during REPRISE and OLE trials in subjects receiving tolvaptan in both trials suggested that reduced decline of eGFR among subjects in the REPRISE placebo data set in the OLE period (tolvaptan-treated) as compared with that in the REPRISE period (placebo-treated) was not a consequence of an unrelated slower pace of eGFR decline as suggested by data in the Modification of Diet in Renal Disease study.^8^ Finally, changes from the REPRISE baseline during the OLE trial were larger in the REPRISE placebo subjects compared with the matched REPRISE tolvaptan subjects owing to the shorter duration of tolvaptan treatment in the former group, yet the change from REPRISE baseline slopes was comparable and parallel for the 1-year period regardless of previous treatment in REPRISE, indicating tolvaptan treatment effects were maintained in advanced CKD G4 (eGFR 15–29).

### Limitations

A limitation of this trial is the retrospective, *post hoc* design. Although the same subject population was compared between 2 consecutive trials and a matched data set was used for further comparison, this analysis was not a randomized, controlled prospective trial. The relatively low number of subjects in this analysis and the high discontinuation rate precluded a safety investigation. Efficacy results are short term owing to the 1-year follow-up of the REPRISE placebo group.

## Conclusions

Results of this *post hoc* analysis suggest that tolvaptan slows eGFR decline in subjects with ADPKD and advanced CKD G4. Treatment with tolvaptan can be initiated or maintained in subjects with ADPKD (CKD G4) once their eGFR drops below 25 ml/min per 1.73 m^2^. Potential benefits and harms of initiating treatment with tolvaptan, such as potential hemodynamic effects for those initiating with GFR 25 to 29, should be discussed with patients in an individualized manner. The cumulative benefit depends on the duration of treatment, which is limited when initiated at an advanced CKD stage. The current eGFR, rate of eGFR decline, estimated time to kidney failure, patient age, and ability to tolerate the medication should all be taken into consideration. In addition, these promising findings should be further investigated in a randomized control trial.

## Disclosure

VET reports receiving grants and/or other fees from Blueprint Medicines, Mironid, Otsuka Pharmaceuticals, Palladio Biosciences, Regulus Therapeutics, and Sanofi-Genzyme, all outside of the submitted work. RTG was a member of the steering committees of the TEMPO, REPRISE, DIPAK-1, and STAGED-PKD studies and received several grants and consultancy fees from Galapagos, Ipsen, Ono Pharma, Otsuka, and Sanofi-Genzyme, in which all money was paid to the employing institution. RDP reports receiving grants from Otsuka; having Otsuka steering committee membership for TEMPO and REPRISE during the conduct of the studies; having membership in the steering committees of the STAGED-PKD and TAME PKD studies; receiving grants from the United States Department of Defense, Kadmon, Reata, and Sanofi-Genzyme; receiving personal fees from Sanofi-Genzyme; and receiving other fees from UpToDate, outside of the submitted work. ABC reports receiving grants from Otsuka; having Otsuka steering committee membership during the conduct of the study; receiving grants and personal fees from Reata and Sanofi-Genzyme; and receiving other fees from UpToDate. JO, JL, HJ, AN, and TW report being employees of Otsuka.

## Supplementary Material

Supplementary File (PDF)

**Table S1**. Incidence of 5 most frequent treatment-emergent adverse events^a^ in any treatment group and hepatic adverse events**.**

**Table S2**. Incidence of serious treatment-emergent adverse events^a^ at frequency of ≥1% in any treatment group.
