# Supplementary material for: Tolvaptan in ADPKD Patients With Very Low Kidney Function
Source: Kidney Int Rep. 2021 Jun 9;6(8):2171–8. doi: 10.1016/j.ekir.2021.05.037 (PMC8343715; doi:10.1016/j.ekir.2021.05.037)
Supplement: Supplementary File (PDF) [file mmc1.pdf]

Table S1. Incidence of five most frequent treatment-emergent adverse events[a] in any treatment group and hepatic adverse events

All subjects with baseline eGFR 15-29			
Adverse event, n (%)	REPRISE placebo subset n=148	REPRISE tolvaptan subset n=148	Total N=296
Thirst	50 (34)	41 (28)	91 (31)
Polyuria	47 (32)	34 (23)	81 (27)
Creatinine increase	35 (24)	36 (24)	71 (24)
Renal pain	39 (26)	25 (17)	64 (22)
Nocturia	34 (23)	23 (16)	57 (19)
Hepatic adverse events			
Alanine aminotransferase abnormal	3 (2.1)	1 (0.7)	4 (1.4)
Aspartate aminotransferase increased	1 (0.7)	1 (0.7)	2 (0.7)
Liver function test abnormal	3 (2.0)	1 (0.7)	4 (1.4)

[a] All adverse events that started after start of trial drug treatment, or if the event was continuous from baseline and was serious, trial drug related, or resulted in death, discontinuation, interruption, or reduction of trial therapy. Subjects are counted once, per term, for the most severe or multiple occurrences of a specific MedDRA Preferred Term. Subjects with adverse events in multiple system organ classes were counted only once towards the total.
eGFR, estimated glomerular filtration rate (in mL/min/1.73 m^2).

Table S2. Incidence of serious treatment-emergent adverse events[a] at frequency of ≥1% in any treatment group

All subjects with baseline eGFR 15-29			
Serious adverse event, n (%)	REPRISE placebo subset<br>n = 148	REPRISE tolvaptan subset<br>n = 148	Total<br>N = 296
Renal impairment	7 (5)	5 (3)	12 (4)
Acute kidney injury	4 (3)	2 (1)	6 (2)
Renal cyst infection	1 (1)	3 (2)	4 (1)
Gastroenteritis	2 (1)	1 (1)	3 (1)
Urinary tract infection	1 (1)	2 (1)	3 (1)
End stage renal disease	0	3 (2)	3 (1)
Hematuria	2 (1)	1 (1)	3 (1)
Renal pain	3 (2)	0	3 (1)

[a]All adverse events that started after start of trial drug treatment, or if the event was continuous from baseline and was serious, trial drug related, or resulted in death, discontinuation, interruption, or reduction of trial therapy. Subjects are counted once, per term, for the most severe or multiple occurrences of a specific MedDRA Preferred Term. Subjects with adverse events in multiple system organ classes were counted only once towards the total.

eGFR, estimated glomerular filtration rate (in mL/min/1.73 m^2).
